# Whole pair distribution function modeling: the bridging of Bragg and Debye scattering theories

**DOI:** 10.1107/S2052252521000324

**Published:** 2021-02-10

**Authors:** Alberto Leonardi

**Affiliations:** a Institute for Multiscale Simulation, IZNF, Friedrich-Alexander-Universität Erlangen-Nürnberg, Cauerstrasse, 3, Erlangen, Bavaria 91052, Germany

**Keywords:** powder scattering, pair distribution functions, Debye scattering equation, line-profile analysis, whole-powder-pattern modeling, Bragg peaks, computing efficiency, common-volume functions

## Abstract

Microstructure-based design of materials requires an atomic level understanding of the mechanisms underlying structure-dependent properties. Methods for analyzing either the traditional diffraction profile or the pair distribution function (PDF) differ in how the information is accessed and in the approximations usually applied. Any variation of structural and microstructural features over the whole sample affects the Bragg peaks as well as any diffuse scattering. Accuracy of characterization relies, therefore, on the reliability of the analysis methods. Methods based on Bragg’s law investigate the diffraction peaks in the intensity plot as distinct pieces of information. This approach reaches a limitation when dealing with disorder scenarios that do not conform to such a peak-by-peak basis. Methods based on the Debye scattering equation (DSE) are, otherwise, well suited to evaluate the scattering from a disordered phase but the structure information is averaged over short-range distances usually accessed by experiments. Moreover, statistical reliability is usually sacrificed to recover some of the computing-efficiency loss compared with traditional line-profile-analysis methods. Here, models based on Bragg’s law are used to facilitate the computation of a whole PDF and then model powder-scattering data *via* the DSE. Models based on Bragg’s law allow the efficient solution of the dispersion of a crystal’s properties in a powder sample with statistical reliability, and the PDF provides the flexibility of the DSE. The whole PDF is decomposed into the independent directional components, and the number of atom pairs separated by a given distance is statistically estimated using the common-volume functions. This approach overcomes the need for an atomistic model of the material sample and the computation of billions of pair distances. The results of this combined method are in agreement with the explicit solution of the DSE although the computing efficiency is comparable with that of methods based on Bragg’s law. Most importantly, the method exploits the strengths and different sensitivities of the Bragg and Debye theories.

## Abstract 2

The whole pair distribution function modeling method is introduced to overcome the distinction between Bragg and Debye theories in analysis of powder-scattering data. Models based on Bragg’s law are used to facilitate the computation of a whole pair distribution function followed by the solution of the Debye scattering equation.

## 1. Introduction   

The synthesis of nanostructured materials with precise control of microstructural properties benefits from the use of powder-scattering methods to fully resolve the structure of the millions to billions of crystals in a typical powder sample (Habas *et al.*, 2007 ▸; Solla-Gullon *et al.*, 2015 ▸; Gamler *et al.*, 2019 ▸; Leonardi & Engel, 2018 ▸). The crystalline structure determines the emergence of diffraction peaks in the scattering profile. Any statistical fluctuation of structural and microstructural properties over the whole sample affects the positions, areas and shapes of the peaks as well as any underlying diffuse scattering (Scherrer, 1918 ▸; Patterson, 1939 ▸). Indeed, microstrain, *i.e.* any distortion of the atomic arrangement as seen through the eye of scattering, broadens and shifts the peak profiles and raises the diffuse component. Different line-profile analysis (LPA) methods have been developed to interpret the two reciprocal representations of powder-scattering data: (i) the traditional diffraction profile, which is the scattered intensity as a function of scattering angle; and (ii) the pair distribution function (PDF), which is the Fourier transform of the scattering data. Although the diffraction profile and its Fourier transform both encode the same information, their analysis is more sensitive to the long- or short-range information, respectively. PDF methods typically resolve the crystal structure within the length scale of a few nanometres, which is currently the experimentally accessible range for most samples (Usher *et al.*, 2018 ▸). Structure distortions over a longer distance range are difficult to extract from the PDF because the information is either not fully accessible or is absent owing to limited experimental resolution. However, PDF methods are capable of resolving local features that are difficult to determine from powder-scattering intensity profiles. The signal from any secondary phase hiding in the diffuse scattering, such as molecules adsorbed at the particle surface, often emerges clearly in the sequence of the PDF peaks. The dependence of the profile broadening on scattering angle or pair distance is characteristic of the distinct source of disorder (Brandstetter *et al.*, 2005 ▸; Ungár *et al.*, 1999 ▸). Thus, accurate materials characterization *via* powder-scattering methods relies on the effectiveness of LPA methods and the reliability of the phenomenological disorder models employed (Scardi *et al.*, 2004 ▸; Wilkens, 1969 ▸, 1970 ▸).

Since 1913, Bragg’s law has been the theoretical model at the base of the analysis of traditional powder-diffraction profiles (Bragg & Bragg, 1913 ▸; Nafday *et al.*, 2018 ▸). Peaks in the intensity plot are investigated as distinct pieces of information (Williamson & Hall, 1953 ▸; Ungár *et al.*, 1998 ▸; Brandstetter *et al.*, 2008 ▸). As an example, although it uses parameters related to the material structural properties, the Rietveld method models the diffraction profiles from powders with a variety of functions that replace the delta lines of Bragg reflections (Rietveld, 1967 ▸, 1969 ▸). The whole-powder-pattern modeling (WPPM) method advances this idea by modeling the peak shape as a function of the crystallite’s microstructure properties (*i.e.* microstrain, crystal size and shape) (Scardi *et al.*, 2000 ▸; Scardi & Leoni, 2002 ▸; Leoni & Scardi, 2004 ▸). Crystalline domain and diffraction-peak shape are linked by the Fourier transform of the common-volume function (CVF), which is calculated for every *hkl* direction reciprocal to the observed *hkl* reflections (Scardi & Leoni, 2001 ▸). Distortion of the atomic arrangement is convoluted to the intensity plot on a peak-by-peak basis (Warren & Averbach, 1950 ▸, 1952 ▸; Warren, 1955 ▸). Hence, the reliability of estimated sample-related parameters is dependent on small perturbations of the crystal structure over the observed sample. Any information hidden in the diffuse scattering component is ignored in favor of high computing efficiency and statistical significance (Coelho, 2018 ▸; Loopstra & Rietveld, 1969 ▸; van Laar & Schenk, 2018 ▸). The Bragg theory is no longer strictly applicable when dealing with complex disorder scenarios, as well as when the variation of the structure factor across the peaks is significant, because changes in the scattering profile do not conform to such a peak-by-peak treatment (Rebuffi *et al.*, 2016 ▸; Leonardi & Bish, 2017 ▸).

Debye tackled the scattering from an ideally disordered amorphous phase in 1915 and PDF methods emerged through the solution of the Debye scattering equation (DSE) (Debye, 1915 ▸). Debye theory can be used to solve the crystal structures of complex molecules or the local distortions in crystalline materials (McGreevy, 1995 ▸; Billinge & Egami, 1993 ▸; Keen, 2001 ▸). The theory provides an estimate of the atomistic model of a crystal explicitly, including the existence of defects and symmetry-dependent structure discontinuities or chemical inhomogeneities. However, statistical reliability and computing efficiency are generally sacrificed to access the information contained in the diffuse scattering component. A single or limited set of atomistic models is used to describe the powder sample to reduce the computing time required for the solution of the PDF. Nonetheless, PDF computation, even for a single model, is significantly slower than the modeling of the reciprocal scattering profile with methods based on Bragg’s law. The reliability of the resulting model is dependent on the uniformity of the crystals in the sample. Indeed, the dispersion of structural and microstructural properties in a sample are both cast in the necessarily limited set of atomistic models, which is used to describe a sample of billions of crystals. Hence, although theoretically possible, the PDF methods usually do not accurately resolve long-range information. PDF methods reach their application limit when dealing with the dispersion of properties over entire powder specimens. In particular, the short-range pair distances usually accessed by experiments are most affected by the larger crystals. Indeed, powder data provide a volume-weighted average of the scattering from the observed crystals.

Neither the PDF nor the methods based on Bragg’s law are fully suited to resolve structure features in both the short and long range, and particularly in the transition regime between these two ranges. To address such limitations, recent studies exploited the Debye theory directly to model the powder-scattering profile from a collection of atomistic models of materials (Cervellino *et al.*, 2003 ▸, 2010 ▸; Scardi & Gelisio, 2016 ▸; Bertolotti *et al.*, 2016 ▸). Such approaches are limited by the intensive computations required. Although a limited set of models is tested, it is possible to use an overabundance of free parameters to describe complex disorder scenarios. Indeed, the greater flexibility of these recent approaches compared with other more traditional approaches (*e.g.* WPPM or Rietveld-like methods) lies in the possibility of adjusting every positional coordinate of each atom in the set of the material model to optimize the agreement between calculated and measured scattering profiles. However, the scattering profile is modeled ignoring the different nature of the information carried by Bragg reflections and diffuse scattering.

Analysis of complex nanostructured materials requires overcoming the separation between Bragg and Debye theories while exploiting their advantages. Herein, we introduce the whole pair distribution function modeling (WPDFM) method, which directly targets the intermediate regime in nanostructured materials. We use models based on Bragg’s law to simplify the computation of a whole PDF and then model scattering data *via* the DSE. Our motivation is twofold. First, models based on Bragg’s law allow for an efficient solution of the dispersion of the crystal’s properties in a powder sample with statistical significance. Second, the mean of the PDF facilitates exploitation of the flexibility of the Debye theory. In other words, PDF methods provide poor information on statistical properties, whereas Bragg’s law approaches are not suitable for providing local information. We compute the intensity profile combining the contributions from every independent crystallographic direction, which are computed by solving the DSE for the associated directional PDFs (D-PDFs). This approach avoids the need for an atomistic model of materials and the need for computation of billions of pair distances. The PDF is not the result of an arbitrary choice of sites out of an infinite lattice and it encompasses every possible arrangement of sites that is compatible with the microstructure properties of interest. In addition to providing results that are in perfect agreement with the explicit solution of the DSE, this method allows high computational efficiency compared with the full solution of the DSE while improving statistical significance.

## 2. Theory   

### 2.1. Modeling the powder-scattering data   

**Figure 1:**
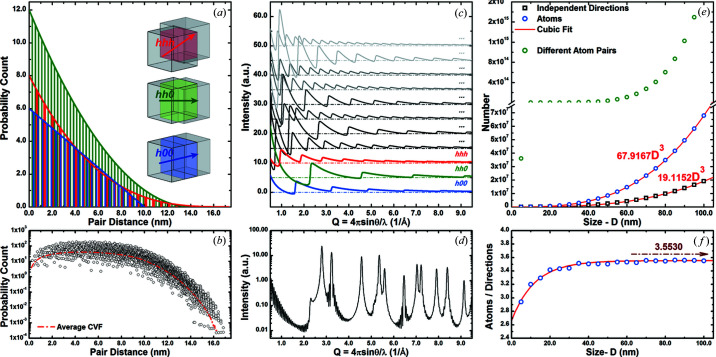
Modeling of the scattering profiles from a Pad (feck) cubic nanocrystal. (*a*) *h*00, *hh*0 and *hhh* D-PDFs estimated from the CVFs. The probability count is normalized by the number of atoms in the crystal to the crystallography redundancy. (*b*) The whole PDF resulting from the superposition of all the independent directional components that relate at least one pair of occupied sites within the crystal. (*c*) Directional components of the Bragg intensity profile computed by solving the DSE for the different D-PDFs. (*d*) The same Bragg intensity profile was computed either by solving the DSE for the whole PDF or by summing the directional components. (*e*) Problem complexity as a function of the crystal size. (*f*) The relation between the number of atoms and the number of independent directions as a function of the crystal size.

The elastic and coherent intensity scattered by a set of *N* atoms of positions *r*_*i* ∈ (0…*N*)_ is
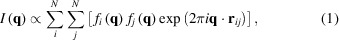
where **q** is the momentum-transfer vector, *f* is the atomic scattering factor, and **r**_*ij*_ = **r**_*j*_ − **r**_*i*_ is the pair-distance vector between atoms *i* and *j*. In a powder, every pair-distance vector is uniformly observed with any possible orientation in space. After integration over the Ewald sphere, equation (1) can be expressed as a function of the momentum-transfer module *Q* = 2π|**q**| as
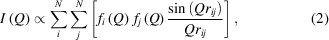
which is the most common formulation of the DSE. It is worth noting that the DSE assumes an ideal sample of equal-sized and equally shaped non-interfering randomly oriented particles, each including *N* atoms in the same configuration. Equation (2) computes the intensity scattered as a function of the set Ω of pair distances *r*_*ij*_ = |*r*_*ij*_| at *Q* = 4π sin θ/λ, where θ is half of the scattering angle and λ is the radiation wavelength. To solve the DSE, a more efficient strategy than brute-force calculation is to use the frequency count *C*_α, β_(ρ_*t*_) of the different pair distances ρ_*t*_ ∈ Ω as
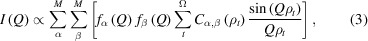
where α and β are the *M* different elemental species in the sample. To deal with a dispersion of numerous crystals, the frequency count is replaced by the probability density, *W*, to find a pair of atoms separated by a given pair distance in the whole sample. Because the sampling of pair distances in a histogram with finite constant step interval, Δ, yields truncation errors (Hall & Monot, 1991 ▸), the PDF is corrected by dynamically shifting the histogram bin centers, γ_*k*_, to suitable magnitudes: η_*k*_ = γ_*k*_ + ξ_*k*_ [Fig. 1 ▸(*b*)]. Although the PDF is recorded using a constant step interval histogram, effective shifts are calculated from the average pair-distance error of each histogram bin as
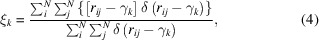
where
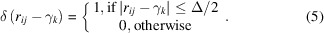
The accurate intensity profile is then calculated as [Fig. 1 ▸(*d*)]
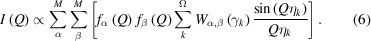
The profile calculated using the corrected PDF is effectively free from any histogram truncation error (Hall & Monot, 1991 ▸; Leonardi & Bish, 2016 ▸). Indeed, our previous study shows that the correction of the PDF bin centers allows the accuracy of the DSE solution in equation (6) to eventually exceed (or at least equal) the accuracy of a brute-force algorithm performed with 64-bit floating-point precision (Leonardi & Bish, 2016 ▸). In contrast to methods based on Bragg’s law, the contribution to the diffuse scattering is fully captured and the structure factor is not approximated to a constant over the scattering-angle range of each diffraction peak.

### 2.2. Modeling the whole pair distribution function   

The modeling of the whole PDF, *W*(γ_*k*_), is decomposed to the calculation of the D-PDF components, ϒ_[*uvw*]_(γ_*k*_), such that
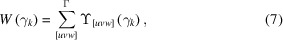
where the set Γ comprises all independent directions [*uvw*] that relate at least one pair of occupied sites within any of the crystal domains in the observed powder sample and |[*uvw*]| = 1. The probability of finding a pair of atoms aligned with any [*uvw*] direction and separated by a pair distance, *L*, can be calculated from atomistic models (Leonardi *et al.*, 2013*a* ▸,*c* ▸). However, here the probability is estimated from the CVF [Fig. 1 ▸(*a*)].

The CVF describes the volume common to a solid shape and a copy of itself translated by a distance *L* along a given direction. As the number of atoms in a crystal is proportional to its volume, with the ratio between the number of atoms in the crystalline structure and the volume of the unit cell being the proportionality constant, the volume common to the two copies of the solid shape is proportional to the number of atom pairs separated by the shapes pair distance (Leonardi *et al.*, 2013*c* ▸). The CVF is independent of the origin of the crystalline lattice relative to the crystal shape. It describes the probability of finding a pair of atoms for the ensemble of all equivalent discrete configurations that fit in the ideal crystal shape (see Section 3.3 for more details). As an example, consider a spherical crystal with a diameter 20 times the unit-cell parameter and simple cubic structure. A discrete model with structure concentric to the shape has a finite integer number of occupied sites (*i.e.* 4169) and first-neighbor pair distances (*i.e.* 23 112), whereas non-integer values are estimated using the sphere volume and the CVF (*i.e.* ∼4188.79 occupied sites and ∼23 249.36 first-neighbor pair distances).

The analytical expression of the CVFs is known for a limited set of shapes, including: sphere, hollow sphere, cube, octahedron, tetrahedron, cylinder and hexagonal prism (see Table S1 in the Supporting information) (Stokes & Wilson, 1942 ▸; Vargas *et al.*, 1983 ▸; Langford & Louër, 1982 ▸; Scardi & Leoni, 2001 ▸; Burresi & Tapfer, 2019 ▸; Leoni, 2019 ▸). However, numerical algorithms can be used to compute the CVF for any arbitrary shape (Leonardi, Leoni, Siboni *et al.*, 2012 ▸). The common volume is calculated at constant pair-distance intervals and for a discrete set of directions. The values are expressed as a function of the normalized pair distance *L*/*D* ≤ 1/*K*_[*uvw*]_, where *D* is a shape-dependent reference size, and 1/*K*_[*uvw*]_ is the critical pair distance at which the unit size solid shape and its copy along the [*uvw*] direction become disjointed. The data for distinct directions can be approximated with piecewise third-order polynomial functions to improve memory and computing efficiency when accessing the information in the following steps. Indeed, either the discrete common-volume values or the coefficients of the polynomial functions are recorded in data tables.

The CVF parameters for any direction of interest [*uvw*] ∈ Γ are interpolated from those tabulated *via* a weighted average. The directions are projected on a unit sphere and the projections of those directions for which the discrete CVFs were recorded are used as tessellation seeds to divide the sphere surface into triangular sectors. The three seeds that define the sector embedding the projection of the direction [*uvw*] are used to identify the set of CVF parameters to average and the barycentric coordinates of the projection of [*uvw*] relative to the seeds are used as weights (see Fig. S1 in the Supporting information). Here we used a Delaunay triangulation, although other triangulations could provide a more accurate approximation for a given crystal shape. Both the critical pair distances and the CVF parameters are interpolated. Whereas the common-volume values are averaged pair distance per pair distance, the polynomial coefficients to average change with the pair-distance intervals that define the piecewise polynomial CVFs. In particular, the set of three piecewise polynomials, defined with two intervals each, average generally into four distinct intervals (Fig. S1). In addition, the CVFs are interpolated between the solutions for stepwise defined classes of similar shapes. As an example, a class of truncated cubic shapes ranging from a cube to an octahedron was defined as a function of the truncation degree of the cube edges and corners (see Section 3.2 for more details). Although the CVFs were calculated with a discrete truncation step interval of 0.5% (*i.e.* 201 solutions), the parameters for any intermediate truncation were estimated *via* linear interpolation. Finally, as it was first proposed for the WPPM method (Scardi & Leoni, 2001 ▸), the CVFs are possibly convoluted with the size probability distribution to describe the dispersion of the crystals in a sample (Fig. S2). In addition, the dispersions of both the crystals’ size and shape are generally described *via* weighted combination of the PDF estimated for a representative population of different crystals (see Section S1 in the Supporting information for more details).

Crystalline materials have a discrete number of atom sites. The CVFs are therefore sampled over a well defined sequence of pair distances (see Section 2.3 for more details). Any distortion of the atomic arrangement will change these distances. The delta lines that characterize the D-PDF profiles of perfect crystals, broaden and shift their centers. Available distortion models from state-of-the-art LPA of either traditional or PDF scattering methods can be readily used to compute the resulting changes. Isotropic and anisotropic thermal vibrations, as well as any available microstrain model, translate directly to the properties (*e.g.* width and skewness) of the peaks’ shape in the D-PDF (Leonardi *et al.*, 2013*a* ▸,*c* ▸; Leonardi & Bish, 2017 ▸; Flor *et al.*, 2019 ▸; Scardi *et al.*, 2017 ▸). As an example, Gaussian peaks with width proportional to the temperature resemble the harmonic oscillator model expressed by the Debye–Waller factor (Debye, 1912 ▸; Waller, 1923 ▸). The Wilkens model for cubic materials (Wilkens, 1969 ▸) and the general model based on the fourth-order invariant introduced by Popa and Adler & Houska (Popa, 1998 ▸; Adler & Houska, 1979 ▸; Leoni *et al.*, 2007 ▸; Martinez-Garcia *et al.*, 2009 ▸) both inherently describe the variation of the D-PDF peaks width as a function of dislocation defects’ type and density (Leonardi, Leoni, Li *et al.*, 2012 ▸; Leonardi *et al.*, 2015 ▸; Leonardi & Scardi, 2015 ▸). The model based on the fourth-order invariant can also be used to describe a wide range of microstrain sources, such as surface relaxation and grain boundaries in polycrystalline materials (Scardi *et al.*, 2015 ▸; Burgess *et al.*, 2013 ▸; Rebuffi *et al.*, 2016 ▸; Leonardi & Bish, 2017 ▸). Indeed, this model does not generally commit to any specific strain model and is capable of capturing the distortion anisotropy. Compared with methods based on Bragg’s law, use of the PDF can easily overcome any assumption often implied by these distortion models, such as the Gaussian-like broadening of the D-PDF peaks (Leonardi *et al.*, 2013*c* ▸). In addition, the broadening and the shift of the centers of the D-PDF peaks can be easily computed from atomistic models of the crystals. The contribution from fault defects can be described either by exploiting the distortion model proposed by Wilson (Wilson & Zsoldos, 1966 ▸; Wilson, 1943 ▸) or by directly computing the change of the pair-distances magnitudes from a structural model of the stacking error (Thomas, 2010 ▸). Features that violate any structural symmetry or periodicity can be included by direct modification of the PDF as carried out with PDF methods. As an example, the distortion field across multi-component nanostructure architectures (Gamler *et al.*, 2020 ▸), as well as the set of pair distances associated with molecules adsorbed at the surface of a nanocrystal, can be computed from atomistic simulations and then included within the estimated PDF.

### 2.3. Determination of base parameters for the directional components   

**Figure 2:**
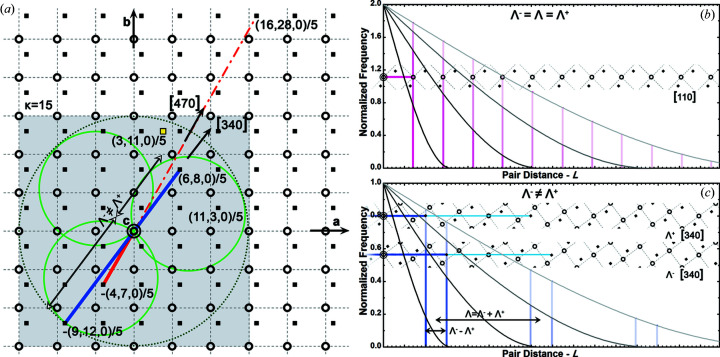
Identification and modeling of the D-PDFs. (*a*) A periodic cubic lattice (black open circles) with an occupied site at relative coordinates (1, 3, 0)/5 (black full squares). The dashed circle is the envelope of any three-unit-cell-diameter spherical region embedding the lattice-origin site. Any direction triplet with a limit index, κ = 15, fits within the gray area. The nearest sites bound along the [340] (blue line) and [470] (red line) directions are shown (see the main text for more details). (*b*) The *hh*0 self-correlation D-PDF estimated from the CVF of four spheres with different diameter sizes. The first non-zero distances to sample equal the separation distances. The normalized frequency is doubled because each pair of sites is observed along the two opposite orientations. (*c*) A cross-correlation D-PDF estimated from the CVF of four spheres with different diameters.

Here we consider the particular case of periodic crystal structures. Constant step intervals, Λ, separate the consecutive pair distances that are to be sampled from the CVF (Fig. 2 ▸). The separation distance is calculated from the distance vector between the nearest pair of atom sites in the crystal, *i* and *j*, aligned with the direction of interest, 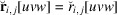, as
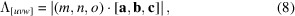
where [**a**, **b**, **c**] is the tensor that defines the crystalline-lattice system and the triplet 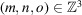 of setwise coprime integers (*i.e.* the greatest common divisor between *m*, *n* and *o* is 1) is such that
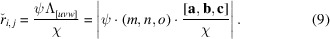
The normalization factor 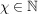 relates the *i*, *j* atoms and their periodic repeats to the nodes of a service lattice homologous to the crystalline-lattice system (*i.e.* [**a**, **b**, **c**]_s_ = [**a**, **b**, **c**]/χ), where 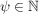 is the number of non-occupied node sites intersected by the distance vector 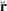 (Fig. S3). Although the first non-zero pair distance to be sampled along [*uvw*] is then
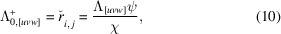
two sequences of equally spaced pair distances are sampled for each direction to account for the opposite orientation pairs: *i* → *j* and *j* → *i* [Fig. 2 ▸(*c*)]. Notably, the first two non-zero pair distances, 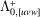 and 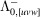, are complementary to the separation distance itself as
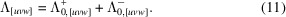
Whereas a bi-modal sequence of pair distances is generally sampled, the correlation of an occupied site with any of its own periodic repeat yields the sampling of a mono-modal sequence with twice the frequency [Fig. 2 ▸(*b*)]. In the case of self-correlating sites, the crystalline and service-lattice systems coincide (χ = 1) and the distance vector 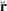 does not intersect any other lattice node besides the first neighbor along the direction [*uvw*] (ψ = 1 or 0). Hence, 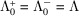 because ψ/χ = 1 or 0. A similar exception is met for the correlation between any periodic repeat of two sites with body-centered symmetry. Indeed, 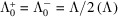 because the symmetry yields χ = 2 and ψ = 1 (1 or 0) for every direction of interest.

The set Γ of independent directions is derived from the set 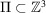 of independent triplets (*h*, *k*, *l*) = ψ(*m*, *n*, *o*), which describe in the service-lattice system any pair of nearest occupied nodes aligned with the direction described by the coprime triplet (*m*, *n*, *o*). It is worth noting that a triplet (*h*, *k*, *l*) is not generally setwise coprime because it can be ψ > 1. As an example, in a cubic lattice with occupied sites of relative coordinates (0, 0, 0) and (1, 3, 0)/5 [Fig. 2 ▸(*a*)], the triplet (6, 8, 0) is not coprime although it relates the origin site with the nearest occupied site aligned with the coprime triplet (3, 4, 0). Indeed, in the service-lattice system with normalization factor χ = 5, the triplet (6, 8, 0) relates the origin with the occupied site (6, 8, 0)/5 = (1, 1, 0) + (1, 3, 0)/5, whereas the site (3, 4, 0)/5 is empty. The set Π is evaluated by testing the condition of independence for any triplet of indices |*h*|, |*k*|, |*l*| ≤ *R*χ = κ that relates a pair of occupied sites, where *R* is the number of unit cells that fit within the size of the modeled crystal. To omit the tedious and computationally expensive test of independence, instead of Π, the set of coprime triplets (*m*, *n*, *o*) is computed. Indeed, they are linearly independent by definition. Coprime triplets are systematically computed using non-intersecting sets of prime factors listed using the sieve of Atkin & Bernstein (2003 ▸). For each coprime triplet, the value of ψ is tested in the range from 1 to κ/max{*m*, *n*, *o*} searching for an (*h*, *k*, *l*) triplet that relates a pair of occupied sites. If no suitable (*h*, *k*, *l*) is found, the coprime triplet is rejected. As an alternative, Π is extracted from an orthogonal three-dimensional bitwise map of the occupied sites. Every location (*h*_*s*_, *k*_*s*_, *l*_*s*_) in the map represents the periodic repeat of one site relative to another one in the base structure unit such that any of the vector pairs expressed in the service lattice are computed as
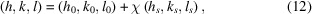
where (*h*_d_, *k*_d_, *l*_d_) is the vector difference between the two sites as they appear within the base structure unit. The locations in the map are systematically explored increasing the indices one at a time from −κ to + κ. For each location explored (*h*_*s*_, *k*_*s*_, *l*_*s*_), the set of locations (*h*_*s*_, *k*_*s*_, *l*_*s*_)′ where
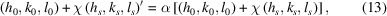
with 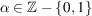, are marked as rejected because they yield linearly dependent (*h*, *k*, *l*) triplets. Whereas those locations marked as rejected are ignored, the others are used to compute the independent triplets that belong to Π [equation (12)]. The coprime triplet (*m*, *n*, *o*) is computed as (*h*, *k*, *l*)/ψ, where ψ is the greatest common divisor between *h, k* and *l*. This second approach is computationally more efficient but its application is limited by the large amount of memory required to record the bitwise map. To improve this approach, the map is divided into Cartesian octants that are explored one next to the other. To avoid the selection of opposite triplets from different octants, additional rejection rules are included, *i.e.* the choice of from which octant a given direction can be extracted.

Different sets Π_*p*_ of independent triplets are evaluated per each correlation of different atom sites from within the base structure unit. Symmetry operations are generally not valid because not every site in the service lattice is occupied by a periodic repeat of either one of the observed atoms. As an example, in the crystalline lattice of Fig. 2 ▸(*a*), although the (11, 3, 0) triplet relates the origin site with the site (11, 3, 0)/5 = (2, 0, 0) + (1, 3, 0)/5, the triplet (3, 11, 0) with swapped indices points to an empty site. Moreover, for each direction, the opposite orientation direction must also be considered. The separation pair distance can be such that the envelope size is exceeded, and only either 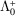 or 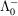 lies within the crystal size. From equations (8), (9), (10) and (11), the directions [*hkl*] and its opposite 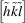 relating the origin to the opposite pair of nearest occupied sites are
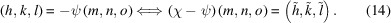
Hence, it is possible that |*h*|, |*k*|, |*l*| ≤ κ while 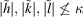. Indeed, in Fig. 2 ▸(*a*) the direction opposite to the triplet (−4, −7, 0) that relates the origin with the site (1, 3, 0)/5 − (1, 2, 0) is the triplet (16, 28, 0), which relates the origin with the site (1, 3, 0)/5 + (3, 5, 0). The positively defined triplet has indices larger than the absolute indices of the negatively defined triplet and it describes a pair distance that would not fit in a crystal with limit index κ = 15 (*i.e.* a three unit-cell-wide envelope region).

**Figure 3:**
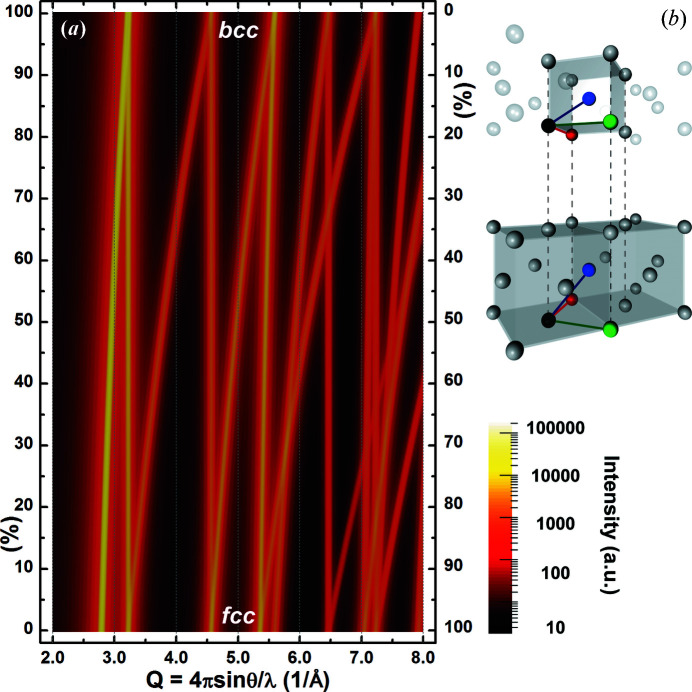
Bain transformation path. (*a*) Intensity-profile evolution for a 10 nm Pd spherical crystal with crystal structure varying between body centered (top) and face centered (bottom). (*b*) End-member structures showing the tensor of the triclinic base system.

The same set Π_0_ is framed by the correlation of any occupied site with its own periodic repeats. The triplets (*h*, *k*, *l*) ∈ Π_0_ are defined in a Cartesian base with no empty lattice nodes regardless of the crystalline-lattice system (whether cubic or not). Thus, the set Π_0_ obeys cubic symmetry. Given the sub-set of independent triplets such that *h* ≥ *k* ≥ *l* ≥ 0, either only positively defined coprime triplets or one-third of the bitwise map for the first octant is explored. The complete set Π_0_ is inferred by the cubic symmetry operations *via* swap of the indices and the indices sign as
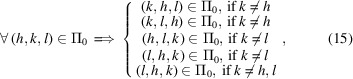
where 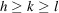 and 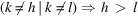 (*i.e.* *h* ≠ *l*), and
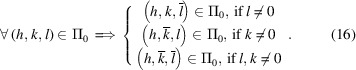
As for the DSE, the WPDFM method disregards the symmetries of the smallest group of particles in the material that constitutes the repeating unit-cell structure. The order imprinted in the sequence of Bragg peaks is ignored. The possible ambiguity of selecting a coherent set of reflections to model the scattering data from highly distorted materials is overcome and any structural phase transformation can be fully resolved (Fig. 3 ▸). The base structure unit is chosen according to the most relevant length scale suitable to capture a material’s feature of interest, and it is iteratively modified to optimize the agreement between observed and modeled scattering data. The base structure unit can be as small as the unit cell of the structure or as large as the entire crystal. Although different sets of D-PDF components and separation distances are estimated, the same whole PDF is computed. Notably, in the extreme case of a large structure unit, the WPDFM would turn into a classical method based on the DSE.

### 2.4. Bravais lattices of monoatomic materials   

Any Bravais lattice of monoatomic materials is described with a primitive triclinic cell through the tensor equivalences of Table 1 ▸. The self-correlation of the cell-origin sites determines the same set of independent directions. Moreover, the CVFs for a powder of spherical crystals are insensitive to the direction of observation. Hence, only the separation distances used to sample the CVFs differentiate the computed whole PDFs. The separation distances change together with the variation of the lattice-system tensor. Different sets of diffraction peaks are suppressed or emerge in the intensity profile. The mean of the whole PDF allows us to model the powder-diffraction profile disregarding the ambiguous task of classifying the local crystal structure and choosing the Bragg components. As an example, crystal systems along the transformation Bain path that connects face-centered and body-centered lattices are continuously modeled as a function of the unit-cell **c** side edge length (Fig. 3 ▸).

## 3. Results and discussions   

### 3.1. Software implementation   

We implemented the WPDFM method within a high-performance computing framework (Fig. S4). The computation of the D-PDFs for the different directions is distributed across the computing processor units (CPUs) with asynchronous parallelization (see Section S2 for more details). The sequence of independent directions is dynamically streamed from a sequential loop, balancing the workflow across multiple thread processes while avoiding the computation of the same direction by multiple tasks. Each thread identifies the next independent direction after locking the loop state. The associated D-PDF component is then computed after unlocking the state. The calculation of the whole PDF by summation of the D-PDFs computed from each thread and the following solution of the DSE are also performed with asynchronous parallelization. Only the sequence of the three stages is synchronized. A significant increase in speed for the solution of the DSE is achieved by exploiting the graphics processing unit (GPU) instead of the CPU. The architecture of a GPU is well suited for the solution of the DSE starting from a PDF. The algorithm is then embedded in an iterative loop to optimize the modeling parameters against the observed scattering profiles. Although we also implemented a simple Monte Carlo method, the parameter optimizations presented here were performed using a simulated annealing scheme.

**Figure 4:**
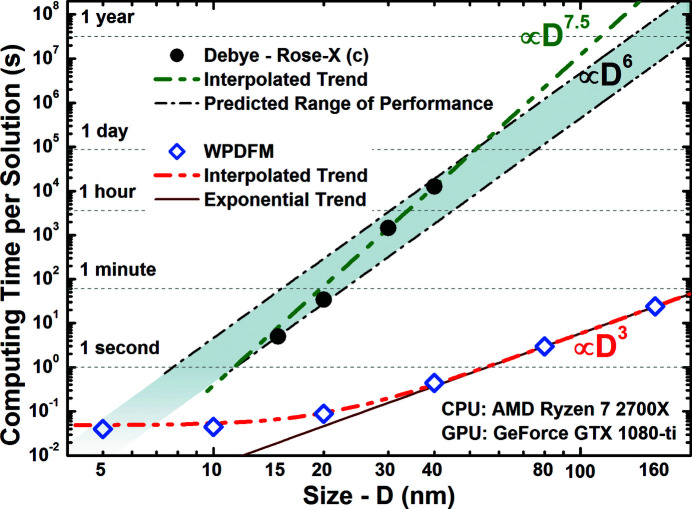
Computing performance. Time to compute a 2000 *Q*-points powder-scattering profile for an f.c.c. Pd nanocube (unit cell = 0.38907 nm) with variable side edge length (*D*). The Debye solution was calculated using the *Rose-X* software application with enabled GPU computing capability. The predicted range of performance of *Rose-X* (shadow region) was estimated based on new calculations and the results reported in Leonardi & Bish (2016 ▸).

Software performance was evaluated against the full solution of the DSE (Fig. 4 ▸) as implemented in the software application *Rose-X* (Leonardi & Bish, 2016 ▸). The performance was measured by the time required to compute a 2000 *Q*-points intensity scattering profile from a powder of ideal face-centred cubic (f.c.c.) Pd nanocrystals (with a unit cell of 0.38907 nm). Although the smallest clock interval that could be recorded was 1 s, the same profile was modeled from scratch 1000 times in order to increase the time resolution to 0.001 s. The performance (computing time) of both methods decreased (increased) with increasing nanocrystal size and the change was proportional to the methods’ complexity. Whereas the number of pair distances accounting for the DSE is known to scale with the square of the crystal volume, the number of independent directions observed for the WPDFM scales linearly with the volume [Fig. 1 ▸(*e*)]. Indeed, the performances of the two methods quickly diverge with an increase of the nanocrystal size. The performance of the WPDFM software deviates from the ideal exponential trend for crystal sizes smaller than 30 nm. Such apparent loss of performance is explained by the fraction of computing time spent to perform memory and initialization tasks that are independent of computing the powder-scattering profile. A similar loss of performance is expected for any software application that exploits parallelization, and in particular GPU capability, to perform a relatively small set of computations. The method based on the DSE is never expected to perform better than the WPDFM method. Indeed, in the limit of a structure-unit-size crystal, the two methods become computationally equivalent because each distinct pair of sites belongs to an independent direction.

### 3.2. Modeling reliability: WPDFM versus Debye   

**Figure 5:**
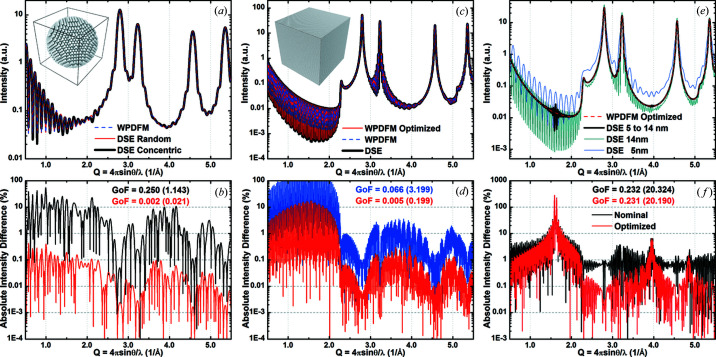
Reliability of the WPDFM method. (*a*), (*c*), (*e*) The intensity profile from Pd crystals with f.c.c. structure (unit cell = 3.8907 Å). (*b*), (*d*), (*f*) Absolute intensity difference between the profiles simulated *via* the DSE and modeled using the WPDFM method. Although the goodness of fit (GoF) of the agreement between the Debye simulated and WPDFM modeled patterns was calculated in the *Q* range from 2.0 to 10.0 Å^−1^, the values calculated including the small-angle region (*Q* ≤ 2.0 Å^−1^) are shown in parentheses. (*a*), (*b*) The intensity profile from a 6 nm spherical Pd (f.c.c.) crystal with perfect structure. The Debye profile is solved for a single configuration with lattice origin at the center of the sphere (concentric), or as the average profile from 1000 equivalent models with a random displacement of the lattice origin. (*c*), (*d*) The intensity profile from a 22 nm cubic Pd (f.c.c.) crystal with perfect structure. The size that optimizes the agreement between Debye simulated and modeled profiles was estimated to be ∼21.98 nm. (*e*), (*f*) The intensity profile from a powder of Pd (f.c.c.) cubic crystals with a uniform size distribution from 5 to 14 nm. The anomalous noise at *Q* ≃ 1.614 Å^−1^ represents the step size of the crystal models that is the Pd unit-cell parameter.

The reliability of the WPDFM method was assessed using virtual experiments (Fig. 5 ▸). Atomistic models of f.c.c. Pd nanocrystals with different shapes were built by selecting from an infinite lattice the sites that are within a given envelope shape (Leonardi *et al.*, 2013*b* ▸). The intensity profile from a powder of perfect crystals was then computed *via* the DSE using the *Rose-X* software (Leonardi & Bish, 2016 ▸). The profiles computed with the WPDFM method and the DSE are in excellent agreement, although they involve different sample approximations.

The CVF provides the statistical probability of observing a pair of atoms at a given distance within the envelope shape regardless of the position of the shape, relative to the lattice origin. Therefore, whereas the DSE provides the intensity profile for a unique configuration of atom sites, the WPDFM inherently describes a set of crystals with the same size and shape [Fig. 5 ▸(*a*)]. To assess this ambiguity, we compared the intensity profile for a spherical crystal computed using the WPDFM method with the DSE solution. We first considered an atomistic model of the spherical crystal with the origin of the lattice origin concentric to the sphere. We then built 1000 different atomistic models of the same spherical crystal randomly displacing the lattice origin relative to the sphere center and computed the average of the corresponding profiles. Compared with the DSE solution for the concentric model, the intensity difference decreased by two orders of magnitude [Fig. 5 ▸(*b*)], supporting the statistical significance of the WPDFM method.

Using a crystal structure and envelope shape with the same symmetry removes the ambiguity of the definition of the atomistic model of the crystal. Except for those site sets that yield the truncation of corners or edges, the same configuration of atom sites is selected by a cubic envelope out of an infinite cubic lattice [Fig. 5 ▸(*c*)]. However, the same sites are also selected by any cubic envelope of size within a unit-cell-wide range [half of it for f.c.c. and body-centred cubic (b.c.c.) crystal structures]. Therefore, in addition to comparing the profiles for the nominal size, we optimized the size used to compute the intensity profile with the WPDFM method against the DSE solution. The best agreement was observed for the average size between those selecting the two atomistic model configurations closer to the nominal cube size. Notably, the intensity difference approached the same value achieved for the spherical crystals after the optimization [Fig. 5 ▸(*d*)].

We further investigated the reliability of the WPDFM in capturing the broadening from the size distribution of crystals in a sample. As the DSE can be solved only for a discrete set of crystal models, as a case study we chose a uniform size distribution of cubic crystals with a side edge length ranging from 5 to 15 nm [Fig. 5 ▸(*e*)]. The independent directional components were estimated using the convolution of the uniform probability distribution of the size with the CVF as
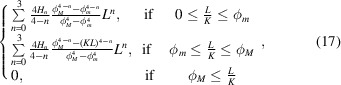
where ϕ_*m*_ ≤ ϕ_*M*_ are the limit sizes and the CVF is described by a third-order polynomial function with coefficients *H*_*n*_. The best agreement between the intensity profiles simulated with the DSE and computed with the WPDFM method was achieved for a size range of about one unit cell wider than expected (*i.e.* from ∼4.8 to ∼15.2 nm). This error pairs with the size step of the atomistic models used to approximate the continuous size distribution in the sample. The high-frequency oscillations from the longer pair distances do not fully cancel because of the finite range and constant step interval of the crystal sizes. Particularly in the small-angle region, the DSE solution shows anomalous deviations from the trend depicted by the intensity profile computed with the WPDFM method. The deviations repeat at constant *Q* intervals of ∼1.61 Å^−1^ corresponding to an inter-planar distance of ∼3.8907 Å, which is the Pd unit-cell parameter [Fig. 5 ▸(*f*)].

### 3.3. Accuracy of modeling using tabulated CVFs   

**Figure 6:**
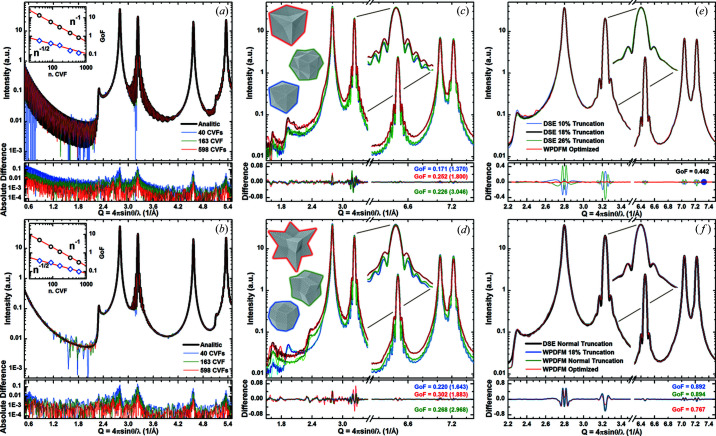
Modeling the scattering profiles from Pd (f.c.c.) nanocrystals with arbitrary shape and shape dispersion. (*a*), (*b*) Intensity profiles from powders of cubic crystals with (*a*) constant and (*b*) lognormal size distribution (mean = 22 nm and s.d. = 1 nm), which were modeled using an increasing number of tabulated CVFs. The profiles are compared against the solution using the analytical CVF expressions. The insets show the GoF of the agreement between the patterns modeled using the analytical and the tabulated CVFs calculated in the *Q* range from 2.0 to 10.0 Å^−1^ (blue, open diamond) or including the small-angle region for *Q* ≤ 2.0 Å^−1^ (black, open circle). The exponential fits are also shown (red, line). (*c*), (*d*) Intensity profiles for powders of polyhedral crystals with the same volume (*i.e.* about the same number of atoms) bounded only by (*c*) 310 and (*d*) 411 facets, which were modeled using 163 tabulated CVFs. (*e*), (*f*) Intensity profiles from powders of truncated cubic crystals with lognormal size distribution (mean ≃ 14 nm and s.d. = 2.2 nm), and (*e*) constant and (*f*) normal truncation distribution (mean = 18% and s.d. = 4%). The profiles are compared with the Debye solution and the GoF of the agreement was calculated in the *Q* range from 2.0 to 10.0 Å^−1^ [the values calculated including the small-angle region (*Q* ≤ 2.0 Å^−1^) are shown in parentheses].

The accuracy of modeling the scattering profile from a powder of shaped crystals is dependent on the accuracy of the CVFs (Fig. 6 ▸). We compared the profiles from powders of cubic crystals modeled using the analytical CVF expressions with those modeled using the tabulated CVF coefficients. The CVF coefficients were calculated for a finite set of independent directions and pair distances (2000 data per direction). We chose the sets of directions to encompass any positively defined independent direction described by a triplet of integers with none of the indices larger than 6, 7, 9, 12 and 15. Therefore, each of these sets, of 40, 88, 163, 334 and 598 directions, respectively, includes those with a smaller threshold index. The difference between the intensity profiles calculated using the analytical and the tabulated CVF decreases exponentially with increasing size of the set of directions [Fig. 6 ▸(*a*)]. Notably, using the largest set, the difference with the solution employing the analytical CVF expressions becomes comparable with the difference between this latter and the solution of the DSE (*i.e.* ∼1.8 10^−3^). The accuracy increases by assuming a dispersion of the crystal sizes [Fig. 6 ▸(*b*)]. For a uniform distribution with an s.d. of 1 nm, the difference between using the analytical or the tabulated CVF decreases by half compared with the monodisperse case. In addition, the improvement becomes greater as the size distribution becomes wider and less discontinuous.

We computed a library of tabulated CVFs for a wide set of polyhedra (Fig. S5). The intensity profiles from powders of identical crystals with envelope shapes of non-regular polyhedra were modeled to assess the contribution of the tessellation used for the interpolation of the tabulated CVFs. Polyhedra bounded by facets from the same family of planes, either 310 [Fig. 6 ▸(*c*)] or 411 [Fig. 6 ▸(*d*)], were chosen here to observe the effect of a different amplitude of edge angles and of going from a convex to a concave shape. The profiles modeled with the WPDFM method using 163 tabulated CVFs are in agreement with the profiles simulated using the DSE. A similar intensity difference with the DSE solution is observed for the profiles from either non-regular polyhedral shape or cubic crystals. Fine features associated with the crystal shape, such as the ‘teeth’ emerging from the low-angle tails of some diffraction peaks, are accurately captured.

To test the accuracy of the new method against state-of-the-art results, we analyzed an intensity profile simulated *via* the DSE for a powder of truncated-cube crystals with lognormal size distribution (mean = 14 nm, s.d. = 2.2 nm) and a constant degree of truncation (18%), reproducing the sample discussed in Scardi *et al.* (2015 ▸) (Scardi & Gelisio, 2016 ▸). Although the intensity variation associated with a truncation degree ranging from 10 to 26% is small, we estimated the expected size-distribution parameters and truncation of 18% with an error of ∼0.0045%, which falls in the range of accuracy of the DSE simulation [Fig. 6 ▸(*e*)]. Improving from previous studies, we also considered the case of simultaneous dispersion of size and truncation degree of crystals. We simulated a normal distribution of the truncation degree with a mean of 18% and an s.d. of 4% for every size fraction in the sample [Fig. 6 ▸(*f*)]. Although the difference between the profiles modeled for a constant or a normally distributed degree of truncation is hidden in minor features, we were able to estimate the expected distribution parameters of both size and truncation degree. Only the mean degree of truncation was slightly underestimated as ∼15.5 instead of 18%. However, this can be attributed to the stepwise approximation of the truncation of the cubic models used to compute the Debye solution.

### 3.4. Polyatomic crystal structures   

**Figure 7:**
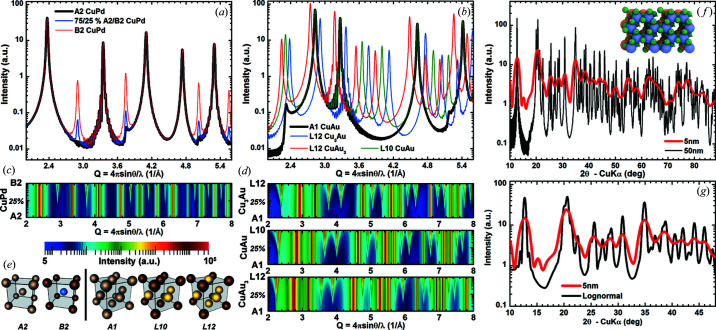
Modeling the scattering profiles from polyatomic nanocrystals. (*a*)–(*e*) Disorder-to-order phase transition of (*a*) and (*c*) b.c.c. Pd–Cu and (*b*) and (*d*) f.c.c. Au–Cu. (*a*), (*b*) Intensity profiles from powders of random alloy and intermetallic 22 nm cubic nanocrystals. (*c*), (*d*) Intensity-profile transformation as a function of the fraction of random *versus* ordered species in the unit structure (the transition is plotted using a quadratic scale). Note that the unit-cell parameter was fixed over the phase transformation. (*e*) Atom-species arrangement in the random alloy and intermetallic end-members crystal structures. (*f*), (*g*) Intensity profiles from powders of kaolinite nanocrystals with hexagonal-prism shape and height equal to the side-to-side distance. Profiles from powders with (*f*) monodisperse crystal size are compared with a powder with (*g*) lognormal size distribution (mean = 15 nm and s.d. = 2 nm).

The scattering profiles from powders of polyatomic crystals are easily modeled using the WPDFM method (Fig. 7 ▸). We observed the intensity profile evolving during the disorder-to-order phase transition in bimetallic cubic crystal structures [Figs. 7 ▸(*a*)–7 ▸(*d*)]. Both A2 Cu–Pd and A1 Cu–Au alloy phases transform to the ordered intermetallic phases, B2 and L10 or L12 for Cu_3_Au and CuAu_3_, whereas they retain the b.c.c. and f.c.c. symmetry of the atom sites, respectively [Fig. 7 ▸(*e*)]. The diffraction peaks observed in the intensity plot from the alloy phase do not change with the phase transformation. In accordance with the WPPM theory, the shape and width are dependent only on the crystal shape and size, which remain the same over the phase transformation. The transformation to the intermetallic phases yields the sequence of reflections characteristic for a simple cubic structure. The reflections forbidden for the alloy phases quickly rise out of the diffuse component with the reordering of the elemental species. Indeed, they are easily distinguishable already with a 25% order [Fig. 7 ▸(*a*)]. The low-angle teeth in the intensity profiles for the alloy phases, as well as for the monoatomic structures, mark the forbidden reflections for both the b.c.c. and the f.c.c. symmetries [Figs. 7 ▸(*a*) and 7 ▸(*b*)]. Although the teeth can be explained by the systematic lack of destructive-interference contributions over the long range, they can also be interpreted as the result of the ambiguous definition of the surface local structure. Atom sites at the surface of a crystal bounded by flat facets show different coordination towards the inside than the outside of the crystal: b.c.c. or f.c.c. inward and simple cubic outward. Whereas the intensity of the reflections allowed by the b.c.c. or f.c.c. symmetries is proportional to the number of atoms in the crystal, the intensity of the teeth is proportional to the number of atoms at the surface. Indeed, the smaller the crystal, the more pronounced the teeth.

Kaolinite crystals were chosen as a case study of a material with complex polyatomic (*i.e.* Al_2_Si_2_O_5_(OH)_4_) and non-cubic crystal structure. Fig. 7 ▸(*f*) shows the scattering profile modeled for powders of hexagonal-prism crystals with height equal to the distance between the pairs of parallel hexagonal sides. We used the crystal structure from Bish (1989 ▸) with C1 symmetry and triclinic unit cell. More than 100 diffraction peaks can be distinguished in the scattering profile from a powder of 50 nm size crystals, in the usual range of measurement for a laboratory diffractometer with Cu radiation. The apparent peak shape is affected by the overlapping of the tails of neighboring peaks. Hence, the accuracy of LPA is affected by the variation of the scattering structure factor for each diffraction peak over the angular region where the individual contribution to the total scattered intensity is not negligible. The DSE is superior to the methods based on Bragg’s law and fully captures these features, but its application is largely limited because the solution for crystals with a size (*e.g.* layer diameter and stacking height) from 50 to 100 nm or more requires very long computation times (*i.e.* from ∼17 h to ∼45 days or more using a desktop computer with an AMD Ryzen 7 2700X CPU and a GeForce GTX 1080-ti GPU). The wide dispersion of sizes in natural samples and the need to decouple the layer diameter from the number of stacked layers makes the solution even more computationally intensive (Leonardi & Bish, 2020 ▸). The WPDFM method provides a very efficient solution compared with the DSE (*e.g.* 261 s for a 50 nm size crystal), at the same time correcting for the change in scattering factor angle-by-angle instead of on a reflection-by-reflection basis as usually carried out with methods based on Bragg’s law. Moreover, the size of the larger crystals in the sample determines the time required to model the scattering profile from a powder sample regardless of the size dispersion. We spent almost the same computing time, ∼58 s, to model the profiles from a powder of 20 nm crystals as that from a powder of crystals with lognormally distributed sizes ranging from 8 to 23 nm [Fig. 7 ▸(*g*)].

## 4. Conclusions   

The Bragg and Debye scattering theories are bridged by WPDFM. Models based on Bragg’s law of material microstructure were used to estimate the whole PDF, which was then employed to solve the DSE. The WPDFM achieved the same accuracy as the DSE solution in modeling the diffuse scattering component and the fine features in the diffraction profiles. Compared with a full solution of the DSE, it is significantly more efficient and inherently captures the statistical nature of a powder. Contrary to methods based on Bragg’s law, the WPDFM method does not rely on the separation of the contributions of the different diffraction reflections to model the line profile. Whether or not the crystalline symmetry is retained, the unit structure used for modeling the scattering profile can be arbitrarily re-defined by adjusting the individual atom sites. Any suitable cluster of the reference crystal unit structure can be used to balance the LPA in the long and short range. Eventually, the unit structure can be optimized, as carried out by other full PDF methods. Disorder models employed by WPPM methods can be directly included and extended. As the PDF describes a sample in real space, detailed material features can be captured by modifying the estimated PDF. As an example, the contribution of polymer molecules bonded at the surface of crystals can be included by adjusting the length of a sub-set of the estimated pair distances and adding those associated with the molecules themselves. Following this, we envision the opportunity to directly employ atomistic simulation to the analysis of scattering data.

The WPDFM method was implemented in a tool for analysis of scattering data, which employs a simulated annealing algorithm to optimize the agreement between observed and modeled profiles. The method was tested against virtual experiments for a wide range of powders of crystals with different structure symmetry, elemental composition, and shape and size dispersions. The contribution of the approximation of using a limited set of tabulated common-volume coefficients instead of analytical expressions was investigated. The scattering profile modeled for powders of polyhedral crystals agreed with the solution of the DSE, regardless of whether the shape was convex or concave. A library of tabulated CVFs was computed for several non-regular polyhedral shapes (avaliable on request). Extending what was carried out with the WPPM method, both the size and truncation-degree dispersions of a powder of Pd truncated-cube crystals, reproducing a real sample that we investigated in the past, were accurately estimated. Finally, intensity profiles from powders of kaolinite crystals and those occurring during structure symmetry or order–disorder transitions of cubic phases were modeled to test the WPDFM with non-cubic and polyatomic crystal structures.

These results suggest that the WPDFM method can overcome current limitations in methods based either on Bragg’s law or the DSE for the analysis of powder-scattering data. Additional work remains to include available disorder models and instrumental contributions before fully exploiting the new environment to strengthen the capability of capturing complex disorder in multicomponent nanostructured materials from their scattering data.

## Supplementary Material

Supporting information. DOI: 10.1107/S2052252521000324/fc5048sup1.pdf

## Figures and Tables

**Table 1:** Equivalence of the system tensors for the Bravais lattices

Primitive	Base centered	Body centered	Face centered
**a**_P_	**a**_C_	**a**_I_	(**b**_F_ + **c**_F_)/2
**b**_P_	(**a**_C_ + **b**_C_)/2	**a**_I_	(**a**_F_ + **c**_F_)/2
**c**_P_	**c**_C_	(**a**_I_ + **b**_I_ + **c**_I_)/2	(**a**_F_ + **b**_F_)/2
