# Supplementary material for: Whole pair distribution function modeling: the bridging of Bragg and Debye scattering theories
Source: IUCrJ. 2021 Feb 10;8(Pt 2):257–69. doi: 10.1107/S2052252521000324 (PMC7924235; doi:10.1107/S2052252521000324)
Supplement: Supplementary file 1 [file m-08-00257-sup1.pdf]

IUCrJ

Volume 8 (2021)

Supporting information for article:

Whole Pair Distribution Function Modeling: the Bridging of Bragg and Debye Scattering Theories

Alberto Leonardi

IUCrJ
ISSN 2052-2525
MATERIALS | COMPUTATION

Whole pair distribution function modeling: the bridging of Bragg and Debye scattering theories

Alberto Leonardi*

Institute for Multiscale Simulation, IZNF, Friedrich-Alexander-Universität Erlangen-Nürnberg Cauerstrasse, 3, Erlangen, Bavaria 91052, Germany. *Correspondence e-mail: alberto.leonardi@fau.de

Keywords: powder scattering; pair distribution functions; Debye Scattering equation; line profile analysis; whole-powder-pattern modeling; Bragg peaks; computing efficiency; common-volume functions.

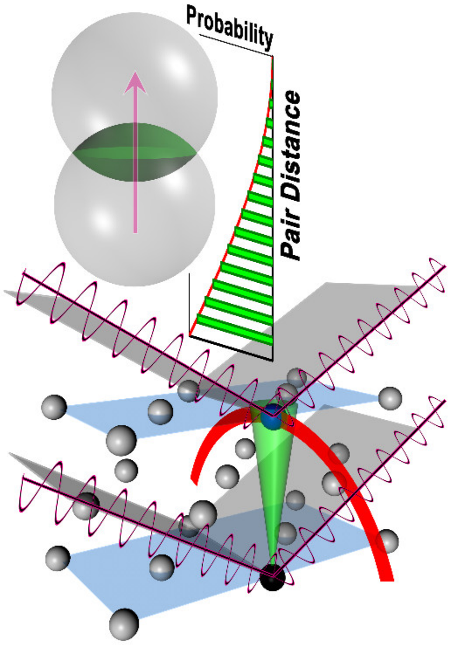

Supporting information

S1. Crystal shape and size dispersions

The dispersion of the crystal shapes and sizes is captured via combination of the contributions from the different sample fractions. For any given crystal with shape σ, the common volume function (CVF), $V_\sigma(L,D)$, is normalized with the shape volume $\forall_\sigma$, such that $V_\sigma(L=0,D)=1$, where L is the pair-distance, and D is the size of the crystal. Therefore, besides to account for the probability of observing a crystal with a given shape and size, $p_S(D)$, the contributions to the pair distribution function are weighted with the reciprocal of the crystal volumes as,

$$\mathrm{V}(L)=\frac{\sum_\sigma\sum_D \mathrm{V}_\sigma(L,D)\xi_\sigma D^3 p_\sigma(D)}{\sum_\sigma\sum_D \xi_\sigma D^3 p_\sigma(D)}, \tag{S1}$$

where $\xi_\sigma = \forall_\sigma/D^3$ is a constant dependent on the crystal shape.
The size-distribution can be convoluted with the CVF for any given shape as,

$$\mathrm{V}(L)=\frac{\sum_\sigma \xi_\sigma \int_0^\infty \mathrm{V}_\sigma(L,D) D^3 g_\sigma(D)\partial D}{\sum_\sigma \xi_\sigma \int_0^\infty D^3 g_\sigma(D)\partial D}, \tag{S2}$$

where $g_\sigma(D)$ is the probability distribution function (Figure S2). To facilitate the solution of the integrals, the CVF can be approximated with a polynomial function with coefficients $H_{\sigma,n}$ as,

$$\mathrm{V}_\sigma(L,D)=\begin{cases}\sum_n H_{\sigma,n}\left(\frac{L}{D}\right)^n, \text{if } \frac{L}{D}\le\frac{D}{K}\\ 0 \qquad\qquad\quad, \text{if } \frac{L}{D}\ge\frac{D}{K}\end{cases}. \tag{S3}$$

Given piecewise polynomial functions with two intervals are better suited to approximate the CVF of concave and non-regular shape, Eq. S3 becomes

$$\mathrm{V}_\sigma(L,D)=\begin{cases}\sum_n U_{\sigma,n}\left(\frac{L}{D}\right)^n, \text{if } \frac{L}{D}\le\frac{D}{T}\\ \sum_n Z_{\sigma,n}\left(\frac{L}{D}\right)^n, \text{if } \frac{D}{K}\ge\frac{L}{D}\ge\frac{D}{T},\\ 0 \qquad\qquad\quad, \text{if } \frac{L}{D}\ge\frac{D}{K}\end{cases} \tag{S4}$$

where $U_{\sigma,n}$ and $Z_{\sigma,n}$ are the polynomial coefficients for the two consecutive intervals, and T and Z are characteristic shape constants.

S2. Algorithm parallelization

The modeling of both the intensity profile and the pair distribution function (PDF) is performed through three subsequent computing stages (Figure S4).

S2.1. Estimation of the directional-pair distribution function (D-PDF)

In this stage, synchronous and asynchronous operations are performed by different CPU threads. Child processes synchronize to retrieve the directional parameters from the master process, which computes the independent directions using a serial

algorithm. The child threads compute the D-PDFs without any synchronization, inherently balancing their workload. A pair of per-thread memory arrays are updated with the estimated probability counts and the difference between actual and recorded pair-distances.

S2.2. Solution of the whole pair distribution function (WPDF)

The data collected by each thread in the first stage are collected. The estimated probabilities and pair distance differences are summed. The recorded pair distances are, then, corrected. Asynchronous computing processes perform this task for a different set of pair-distances (i.e., non-empty memory locations).

S2.3. Modeling of the scattering profiles

Experimental-like scattering profiles are computed: the intensity profile via Debye scattering equation (DSE), and the PDF resampling the high accuracy whole PDF. Both the scattering profiles are corrected to match the corresponding experimental representation (e.g., removal of the small-angle contribution to the PDF, and rescaling with the pair-distance).

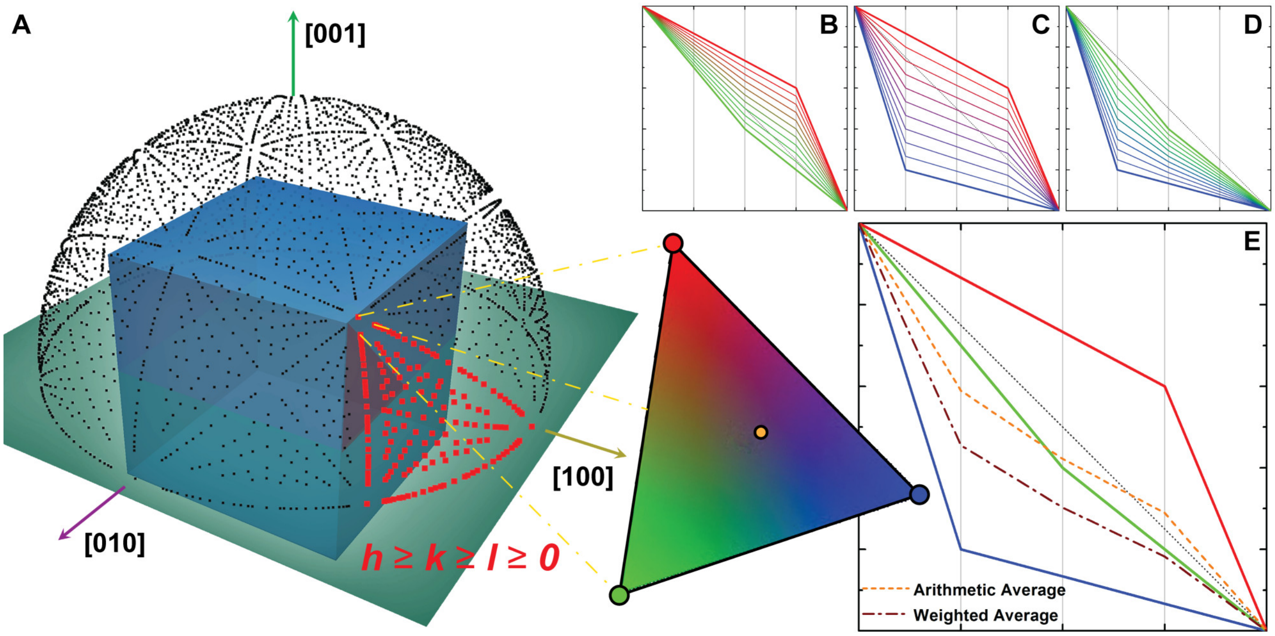

Figure S1 **Interpolation of tabulated common volume functions. A**, projection on a unit-sphere of the directions for which are recorded the CVFs. In case the correlation between a site and its own repetition is considered, the complete set (black dots) is inferred from a subset in the first octant (red dots). A triangular sector from the sphere surface is mapped with an RGB color scheme using the barycentric coordinates as the R, G, and B factors. **B**, **C**, and **D**, variation of the CVF interpolated along the edges of the triangular sector. **E**, Interpolation of the two-intervals piecewise CVFs tabulated for the directions associated with the triangular sector corners. In **B** to **E**, the intervals into which the CVFs are divided are marked (gray line). Note that the CVFs for the sector corners are only example profiles, and they were not computed from any shape.

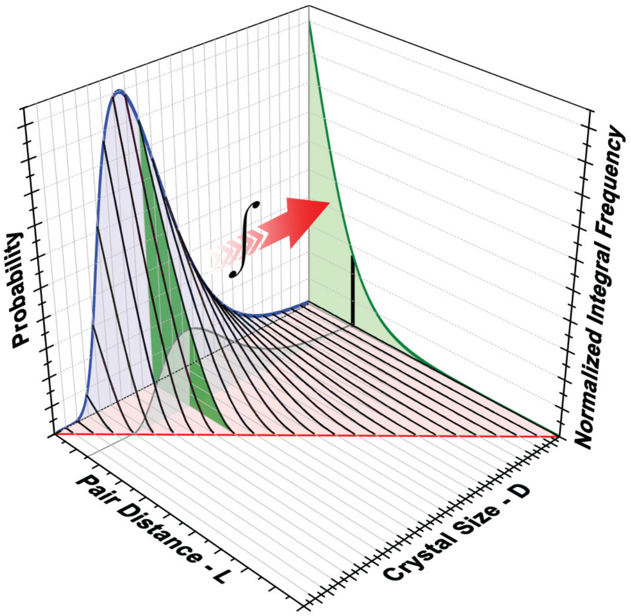

Figure S2 **Graphical representation of the convolution of the CVF with the size dispersion.** The CVF profile (black line) scale with the crystal size (red line) and the size probability distribution function (blue line). The integral frequency for the dispersion of crystals' size (green line) is computed as the area under the cross-section of the CVFs surface for constant pair distance (gray line).

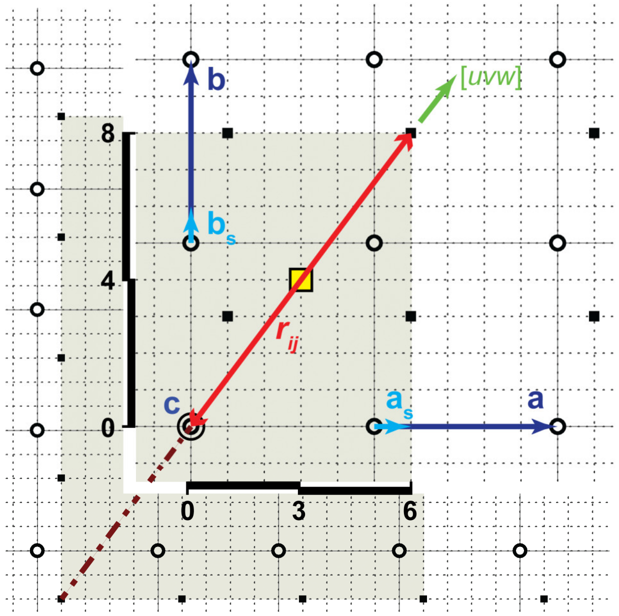

Figure S3 **Site periodicity in the service lattice system.** Cubic crystalline lattice system (continuous gray line) with occupied sites of relative coordinates (0,0,0) and (1,3,0)/5 (black open circle and full square, respectively). Note that the positive quadrant is magnified. All the occupied sites are at the nodes of the service lattice (dotted gray line) of normalization factor $\chi = 5$. The vector pair distance $\mathbf{r}_{ij}$ aligned with the direction $[uvw]$ that binds the origin with the $(6,8,0)/5$ site is shown to intersect also the site $(3,4,0)/5$ that belongs to the service lattice system. The same site type repeat along the [uvw] direction at constant step intervals with coprime triplet (3,4,0) the step interval in the crystalline lattice system.

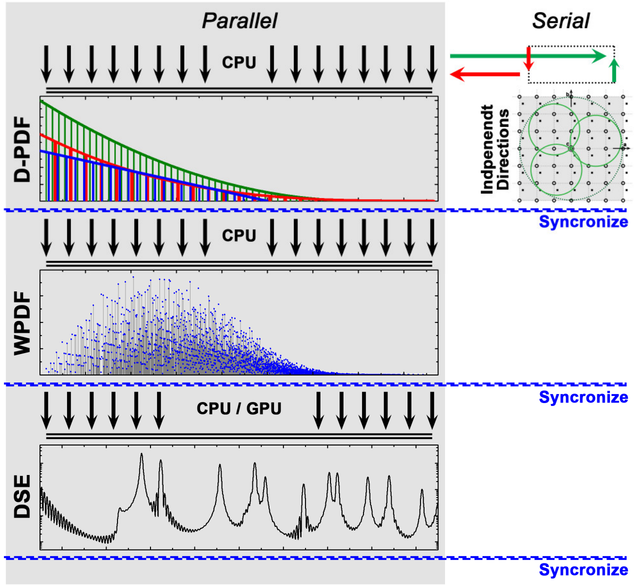

Figure S4 **Algorithm parallelization.** Computing stages used to model both the intensity profile and the pair distribution function (PDF).

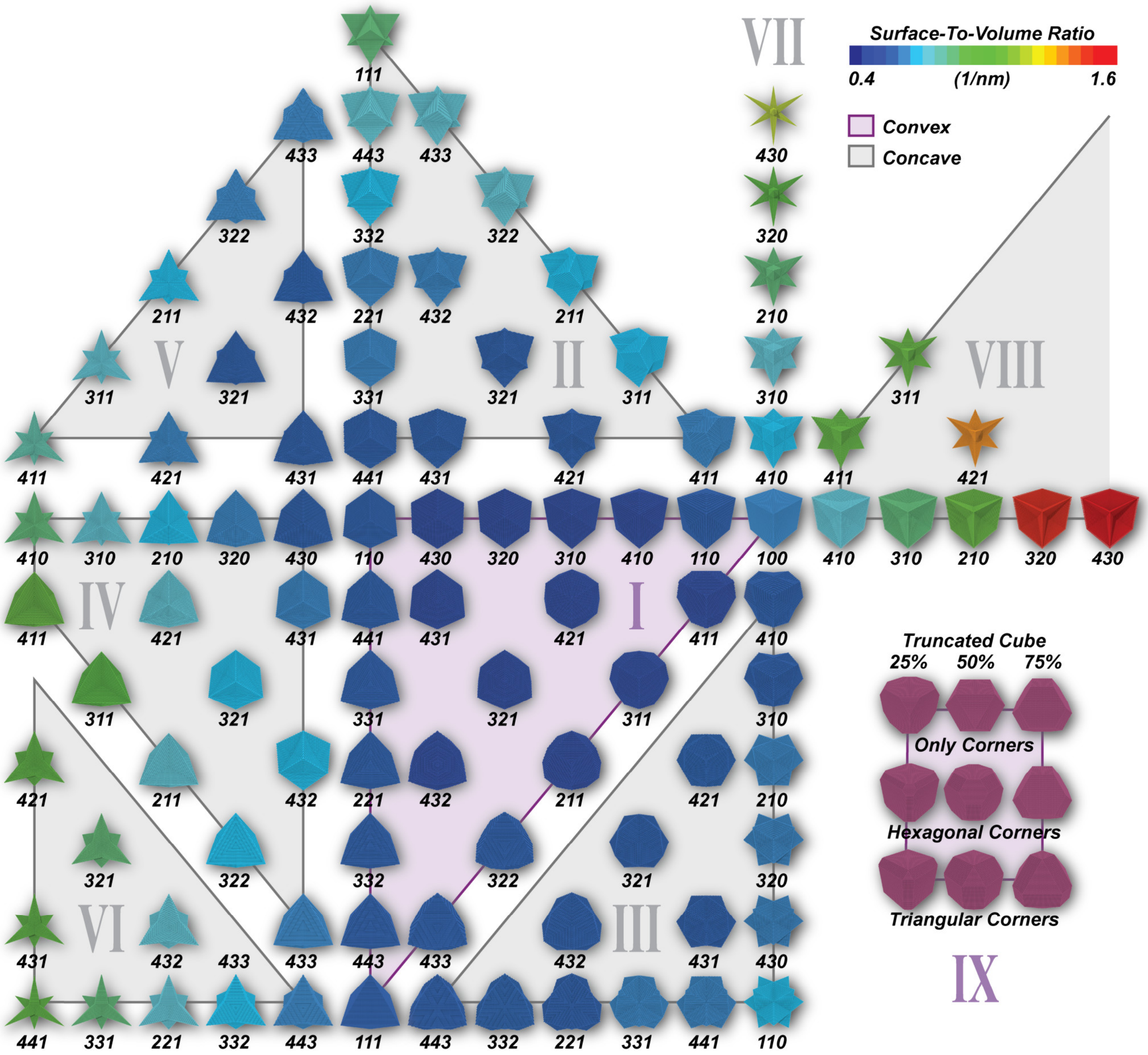

Figure S5 **Map of the library of shapes for which the CVF coefficients were tabulated.** The CVF coefficients were computed for a set of polyhedral shapes bounded by a single family of planes (*hkl* indices in the map) and for a set of truncated cubes with 200 degrees of truncation. The shapes were separated into homogeneous groups (I to IX) according to the set of edges and corners angles. The shapes are colored according to the surface-to-volume ratio (color bar) assuming the polyhedral crystals with volume 10^3 nm^3. The CVF coefficients were calculated for 163 positively defined independent directions described by triplets of integers with none index larger than 9 (2000 data point per direction). The values were interpolated either with a single cubic or a pair of cubic functions. The data were formatted to be compatible with the software package PM2K. Because of size limitation, the complete data set is available on request.

Table S1 **Analytical expression of the common volume functions (CVF).** Unless for the sphere shape, the CVF is a function of the observed direction (A, B, C) positively defined in a Cartesian space such as $\|A, B, C\| = 1$. Whereas for the cube, the tetrahedron, and the octahedron the direction components are reordered such that $A \geq B \geq C \geq 0$, for the cylinder and hexagonal prism C is aligned with the shape axes and B is normal to a pair of opposite hexagonal sides. The cube and octahedron shapes are assumed to be bounded by $\{1,0,0\}$ and $\{1,1,1\}$ planes, respectively. The tetrahedron is assumed to be bounded by the $(1,1,1)$, $(1,\bar{1},\bar{1})$, $(\bar{1},1,\bar{1})$, and $(\bar{1},\bar{1},1)$ planes. Depending on the shape, D is: the diameter of the sphere; the edge length of the cube, the tetrahedron, and the octahedron; the height and the base diameter of the cylinder, or distance between a pair of opposite sides of the hexagonal prism. The equations are written for $0 \leq L \leq D/K$. The CVF for the hexagonal prism split into two pieces at $L = D/K^*$.

Shape	***Case***	***Common Volume Function***	***Boundary (K)***
Sphere	-	$1-\frac{3}{2}\left(\frac{L}{D}\right)+\frac{1}{2}\left(\frac{L}{D}\right)^3$	1
Cube	-	$1-(A+B+C)\left(\frac{L}{D}\right)+(AB+AC+BC)\left(\frac{L}{D}\right)^2-ABC\left(\frac{L}{D}\right)^3$	A
Tetrahedron	$A \geq B+C$	$1-3\sqrt{2}A\left(\frac{L}{D}\right)+6A^2\left(\frac{L}{D}\right)^2-2\sqrt{2}A^3\left(\frac{L}{D}\right)^3$	$\sqrt{2}A$
	$A \leq B+C$	$1-\frac{3(A+B+C)}{\sqrt{2}}\left(\frac{L}{D}\right)+\frac{3(A+B+C)^2}{2}\left(\frac{L}{D}\right)^2-2\sqrt{2}A^3\left(\frac{L}{D}\right)^3$	$\frac{A+B+C}{\sqrt{2}}$
Octahedron	$A \geq B+C$	$1-\frac{3A}{\sqrt{2}}\left(\frac{L}{D}\right)+\frac{3(A-B^2-C^2)}{2}\left(\frac{L}{D}\right)^2-\frac{-A^3+3A(B^2+C^2)+2(B^3+C^3)}{2\sqrt{2}}\left(\frac{L}{D}\right)^3$	$\frac{A+B+C}{\sqrt{2}}$
	$A \leq B+C$	$1-\frac{3(A+B+C)}{2\sqrt{2}}\left(\frac{L}{D}\right)+\frac{-3[A^2+(B-C)^2-2A(B+C)]}{4}\left(\frac{L}{D}\right)^2-\frac{A^3+B^3+C^3-3ABC}{\sqrt{2}}\left(\frac{L}{D}\right)^3$	$\frac{A+B+C}{\sqrt{2}}$
Cylinder	-	$\left[1-C\left(\frac{L}{D}\right)\right]\frac{2}{\pi}\left\{\cos^{-1}\left(\sqrt{A^2+B^2}\left(\frac{L}{D}\right)\right)-\left[\sqrt{A^2+B^2}\left(\frac{L}{D}\right)\right]\sqrt{1-(A^2+B^2)\left(\frac{L}{D}\right)^2}\right\}$	$\max\left\{C,\sqrt{A^2+B^2}\right\}$
Hexagonal Prism	$\begin{cases} A \leq 1 \\ A \geq \frac{\sqrt{3}}{2} \end{cases}$	$\left[1-C\left(\frac{L}{D}\right)\right]\left\{\frac{2}{3}\left[1-B\left(\frac{L}{D}\right)\right]\left[2-(\sqrt{3}A+B)\left(\frac{L}{D}\right)\right]-\frac{4}{3}\left[\frac{1}{2}-B\left(\frac{L}{D}\right)\right]^2\right\}$	$\left(\frac{L}{D}\leq\frac{1}{\sqrt{3}A-B}\right)^*$
		$\left[1-C\left(\frac{L}{D}\right)\right]\left\{\frac{1}{3}\left[2-(\sqrt{3}A+B)\left(\frac{L}{D}\right)\right]\left[2-(\sqrt{3}A-B)\left(\frac{L}{D}\right)\right]\right\}$	$\max\left\{C,\frac{\sqrt{3}A+B}{2}\right\}$
	$\begin{cases} A \leq \frac{\sqrt{3}}{2} \\ A \geq \frac{1}{2} \end{cases}$	$\left[1-C\left(\frac{L}{D}\right)\right]\left\{\frac{2}{3}\left[1-B\left(\frac{L}{D}\right)\right]\left[2-(\sqrt{3}A+B)\left(\frac{L}{D}\right)\right]-\frac{4}{3}\left[\frac{1}{2}-B\left(\frac{L}{D}\right)\right]^2\right\}$	$\left(\frac{L}{D}\leq\frac{1}{B}\right)^*$
		$\left[1-C\left(\frac{L}{D}\right)\right]\left\{\frac{2}{3}\left[1-B\left(\frac{L}{D}\right)\right]\left[2-(\sqrt{3}A+B)\left(\frac{L}{D}\right)\right]\right\}$	$\max\left\{C,\frac{\sqrt{3}A+B}{2}\right\}$
	$\begin{cases} A \leq \frac{1}{2} \\ A \geq 0 \end{cases}$	$\left[1-C\left(\frac{L}{D}\right)\right]\left\{\frac{2}{3}\left[1-B\left(\frac{L}{D}\right)\right]\left[2-(\sqrt{3}A+B)\left(\frac{L}{D}\right)\right]-\frac{1}{3}\left[1-(\sqrt{3}A+B)\left(\frac{L}{D}\right)\right]^2\right\}$	$\left(\frac{L}{D}\leq\frac{1}{\sqrt{3}A+B}\right)^*$
		$\left[1-C\left(\frac{L}{D}\right)\right]\left\{\frac{2}{3}\left[1-B\left(\frac{L}{D}\right)\right]\left[2-(\sqrt{3}A+B)\left(\frac{L}{D}\right)\right]\right\}$	$\max\{C,B\}$
